# Multiple radial artery pseudoaneurysms including an unrelated site of arterial catheterization after transradial coronary intervention: a case report

**DOI:** 10.1093/jscr/rjaf524

**Published:** 2025-07-15

**Authors:** Hee Suk Jung, Kwan Wook Kim

**Affiliations:** Department of Thoracic and Cardiovascular Surgery, CHA Bundang Medical Center, Seongnam-si, South Korea; Department of Thoracic and Cardiovascular Surgery, CHA Bundang Medical Center, Seongnam-si, South Korea

**Keywords:** pseudoaneurysm, percutaneous coronary intervention, radial artery

## Abstract

Radial artery pseudoaneurysm (RAP) is a rare iatrogenic complication associated with percutaneous radial artery catheterization. However, RAP has become more common with the increased use of transradial coronary intervention. RAP has a diverse range of treatment strategies—from conservative management to endovascular treatment and surgery. We report on the case of a 66-year-old male patient with a 1-month prior history of transradial coronary intervention. A computed tomography (CT) scan was performed to evaluate pain, swelling and palpable masses on right forearm. The results of this CT revealed two discrete RAPs. Surgery was performed for complete excision of the RAPs after percutaneous endovascular exclusion using covered stent failed.

## Introduction

The radial artery is a preferred site of percutaneous catheterization for arterial blood sampling, continuous blood pressure monitoring and coronary angiography. It has been shown to have fewer vascular complications with earlier patient ambulation and greater postprocedural comfort. Despite these benefits, transradial access is still associated with vascular complications such as spasm, thrombotic occlusion, hematoma, arteriovenous fistula, compartment syndrome, and pseudoaneurysm [1]. Among these, radial artery pseudoaneurysm (RAP) is extremely rare. The incidence of RAP is estimated to occur in 0.009% of cardiac catheterizations performed through radial artery access [2]. Nonetheless, with the increased use of transradial coronary interventions, RAP has become relatively common [3]. Although RAP treatment strategies have not been well established, the majority of such cases can be managed conservatively. In cases where conservative treatment fails, surgery remains the gold standard and percutaneous endovascular exclusion can be a good alternative [4]. In this study, we report on a notable occurrence of multiple RAPs subsequent to transradial coronary intervention.

## Case report

**Figure 1:**
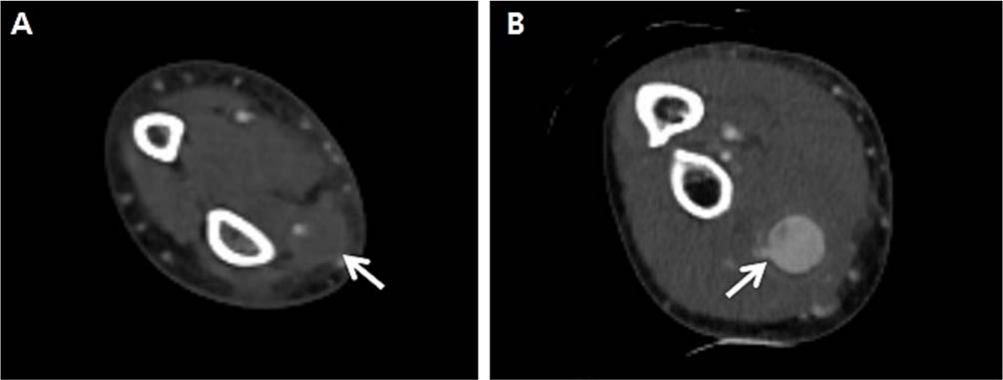
Preprocedural computed tomography of the right upper extremity. (A) Pseudoaneurysm at the distal radial artery which was arterial catheterization site (arrow); (B) Pseudoaneurysm at the proximal radial artery which was not related to arterial catheterization site (arrow).

A 66-year-old male with a 1-month prior history of transradial coronary intervention visited hospital for aggravating pain and swelling on the right forearm. Upon physical exam, multiple masses were palpated at the arterial catheterization site as well as another unrelated site at the proximal forearm. An upper extremity CT scan was conducted to evaluate two different RAPs, one at the distal radial artery (arterial catheterization site) measuring 0.8 cm without a neck of pseudoaneurysm (Fig. 1A) and one at the proximal radial artery (Not related to arterial catheterization site) measuring 2.0 cm with a neck of pseudoaneurysm (Fig. 1B). Blood tests for vasculitis or autoimmune disease are not specific to confirm the possible causes of RAPs.

**Figure 2:**
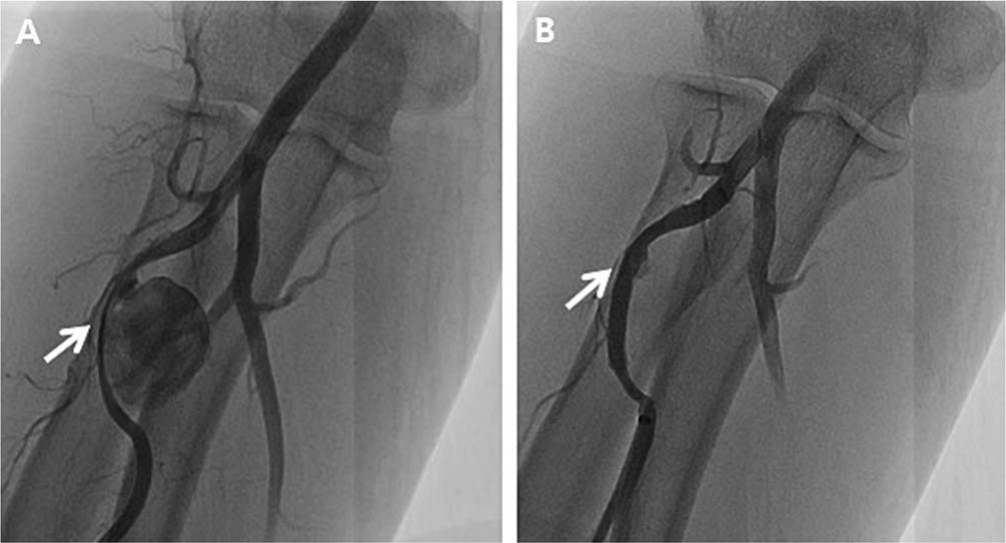
Peripheral angiography of the right upper extremity. (A) Pseudoaneurysm at the proximal radial artery (arrow); (B) Complete occlusion of pseudoaneurysm after covered stent placement (arrow).

**Figure 3:**
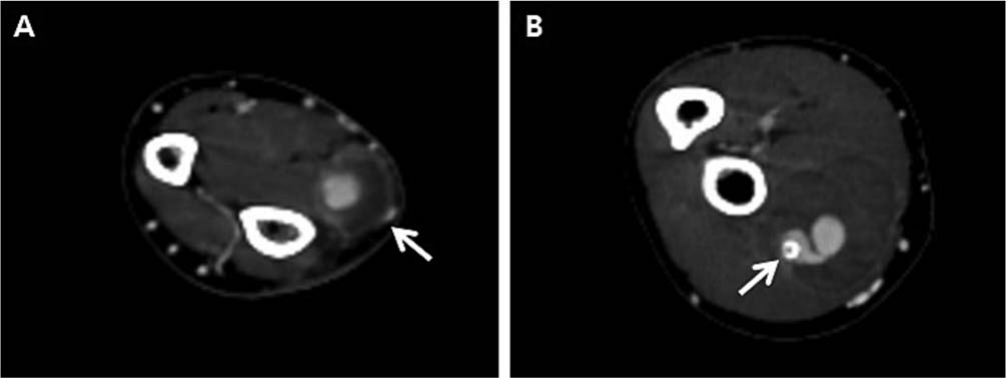
Follow-up computed tomography of the right upper extremity, 2-weeks after the procedure. (A) Pseudoaneurysm at the distal radial artery, size increased from 0.8 cm to 1.5 cm (arrow); (B) Pseudoaneurysm at the proximal radial artery, size increased from 2.0 cm to 4.0 cm with distal migration of covered stent (arrow).

Initially, we conducted percutaneous endovascular exclusion of the proximal RAP using 3.5 × 26 mm Graftmaster covered stent (Abbott Vascular, Santa Clara, CA, USA) and observation of the distal RAP (Fig. 2). Nevertheless, no improvements were observed in the RAPs for 2 weeks after the procedure. A follow-up CT showed the distal migration of the covered stent and increased size of the RAPs. Distal lesion size increased from 0.8 cm to 1.5 cm (Fig. 3A) and the proximal lesion increased from 2.0 cm to 4.0 cm (Fig. 3B). At this point, surgery was considered suitable for the patient to achieve complete recovery.

**Figure 4:**
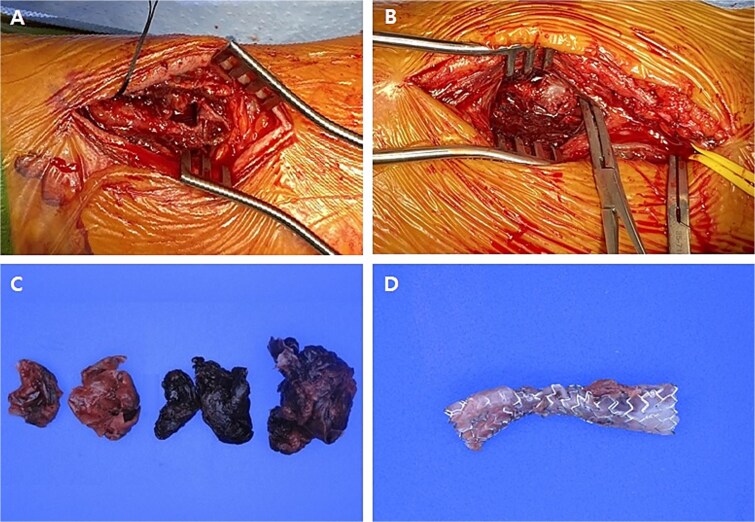
Intraoperative images and pathological specimens after the resection of pseudoaneurysms. (A) Distal lesion which was arterial catheterization site; (B) proximal lesion which was not related to arterial catheterization site; (C) the wall of pseudoaneurysm and hematoma (resected); (D) covered stent (removed).

Pre-operatively, a modified Allen test was performed to verify sufficient blood flow to the hand through the ulnar artery in case of radial artery ligation. As a result, the test was positive indicating the ulnar artery had sufficient blood flow. Although the patient underwent the resection of both RAPs, the severely damaged radial artery was inevitably sacrificed (Fig. 4).

The patient was postoperatively discharged home on day 2 without any complications. At the 1-month follow-up, the patient was asymptomatic and did not report any neurological deficits.

## Discussion

Transradial coronary intervention certainly offers advantages in terms of vascular complication. Some studies have demonstrated the rate of major vascular complications as 0.6%–1.4% [5, 6]. Moreover, RAP after transradial coronary intervention is an extremely rare complication [2, 7].

RAP is caused by a penetrating injury of the arterial wall during catheterization resulting in hemorrhage and hematoma. RAP occurrence is associated with several predisposing factors, such as multiple puncture attempts, systemic anticoagulation, inadequate hemostasis, catheter infection and the use of larger sheath sizes. In the majority of cases, RAP presents with a pulsatile mass, associated with pain and edema [8].

RAP is usually associated with the arterial catheterization site. However, a complication involving a more proximal vessel perforation has also been described [9]. Although it could not be verified, in our case the possible cause of the large RAP at the proximal radial artery was not directly related to the arterial catheterization. Instead, it is thought to be the result of a microperforation of the proximal radial artery during a transradial coronary intervention.

Due to the rare incidence of RAP, there is no uniform consensus in its management strategies. Obviously, early diagnosis and treatment is important to minimize further complications and maintain sufficient hand circulation [10]. Generally, RAP presents as a pulsatile mass with pain, edema and ischemia. Doppler ultrasonography and computed tomography are gold standards to confirm RAP diagnosis.

Conservative management of small-sized, asymptomatic RAPs (<10 mm in diameter), such as ultrasonography, guided compression or minimally invasive thrombin injections have also been employed for treatment [11]. However, compression is usually inefficient in patients taking multiple anti-thrombotic medications [12]. Thrombin injections are also limited to the treatment of narrow neck RAPs and has the risk of distal embolization [13].

Less invasive than surgery, endovascular treatment, such as embolization or endovascular exclusion of RAP with covered stent by retrograde ipsilateral ulnar access or antegrade brachial access can be good alternatives. However, endovascular treatment needs to be further studied to determine its long-term efficacy [14]. Although there is no precise consensus about treatment strategies, surgery is recommended for large RAPs (>10 mm in diameter), fast growing masses, infection and significant mass effects. Surgery is also indicated when conservative treatment or endovascular treatment fails [15]. However, surgery adds further burden to an already comorbid patient for its invasiveness. This is the principle reason that we recommend conservative or endovascular treatment first.

In this case, the percutaneous endovascular exclusion of the RAP with a covered stent had failed. Subsequently, we conducted the surgical resection of two different RAPs and ligated the severely damaged radial artery successfully.

## Conclusion

RAP is a rare condition. Most RAP cases are related to arterial catheterization. However, RAP at an unrelated site of arterial catheterization can occur and it is extremely rare. Careful observation and early diagnosis are essential. Treatment strategies can range from conservative care to surgery depending on the specific presenting characteristics of the RAP.
